# Identifying prodromal symptoms at high specificity for Parkinson’s disease

**DOI:** 10.3389/fnagi.2023.1232387

**Published:** 2023-09-22

**Authors:** Holly Jackson, Judith Anzures-Cabrera, Tanya Simuni, Ronald B. Postuma, Kenneth Marek, Gennaro Pagano

**Affiliations:** ^1^Roche Products Ltd, Welwyn Garden City, United Kingdom; ^2^Department of Mathematics and Statistics, Lancaster University, Lancaster, United Kingdom; ^3^Department of Neurology, Northwestern University Feinberg School of Medicine, Chicago, IL, United States; ^4^Department of Neurology, Montreal Neurological Institute, McGill University, Montreal, QC, Canada; ^5^Institute for Neurodegenerative Disorders, New Haven, CT, United States; ^6^Roche Pharma Research and Early Development (pRED), Neuroscience and Rare Diseases Discovery and Translational Area, Roche Innovation Center Basel, Basel, Switzerland; ^7^University of Exeter Medical School, London, United Kingdom

**Keywords:** Parkinson’s disease, prodromal symptoms, specificity, prevalence, observational study

## Abstract

**Introduction:**

To test drugs with the potential to prevent the onset of Parkinson’s disease (PD), it is key to identify individuals in the general population at high risk of developing PD. This is often difficult because most of the clinical markers are non-specific, common in PD but also common in older adults (e.g., sleep problems).

**Objective:**

We aimed to identify the clinical markers at high specificity for developing PD by comparing individuals with PD or prodromal PD to healthy controls.

**Methods:**

We investigated motor and non-motor symptoms (Movement Disorder Society Unified Parkinson’s Disease Rating Scale Part 1 and 2 items) in 64 prodromal PD and 422 PD individuals calculating the odds ratios, adjusting for age and gender, for PD and prodromal PD versus 195 healthy controls. Symptoms at high specificity were defined as having an adjusted odds ratio ≥ 6.

**Results:**

Constipation had an adjusted odds ratio, 6.14 [95% CI: 2.94–12.80] showing high specificity for prodromal PD, and speech difficulties had an adjusted odds ratio, 9.61 [95% CI: 7.88–48.81] showing high specificity for PD. The proportion of participants showing these specific markers was moderate (e.g., prevalence of constipation was 43.75% in prodromal PD, and speech difficulties was 33.89% in PD), suggesting these symptoms may make robust predictors of prodromal PD and PD, respectively.

**Discussion:**

Clinical markers at high specificity for developing PD could be used as tools in the screening of general populations to identify individuals at higher risk of developing PD.

## 1. Introduction

Parkinson’s disease (PD) diagnosis is clinical, based on the presence of motor features, such as bradykinesia, rigidity and resting tremor (de Lau and Breteler, 2006). Prodromal Parkinson’s disease is a stage of PD wherein neurodegeneration has started but the full motor signs (bradykinesia, rigidity, and resting tremor) are not fully established yet and hence, PD has not been clinically diagnosed (Postuma et al., 2012). Drugs that aim to slow disease progression should be started as soon as neurodegeneration begins, thus the need to identify prodromal PD for future clinical trials.

Currently, prodromal PD is very difficult to diagnose in the general population (Postuma et al., 2016), as there is no 100% reliable, ready and available test to identify this condition (Berg et al., 2014) as no biological definition of PD has been accepted yet. A proposal put forward by the International Parkinson and Movement Disorders Society task force suggested that diagnostic criteria for prodromal PD should be probabilistic and it should include clinical motor and non-motor markers, and non-clinical biomarkers (Berg et al., 2014). A Bayesian method to predict the probability of diagnosis was explored by sequentially adding diagnostic information. The method starts with an initial prior probability of diagnosis, using an age-adjusted prevalence of prodromal PD and this probability is updated using likelihood ratios based on the strength of the diagnostic test (Berg et al., 2015). This method is however difficult to use in clinical practice or in screening of the general population.

In the last ten years, there has been a notable increase in the research into prodromal PD and into the potential markers which could be used in a biological diagnosis of PD (Postuma and Berg, 2019). Several predictive models for PD have been suggested in the last decade, ten of which were critically appraised in a systematic review by Chen et al. (2023). Three models: Mahlknecht et al. (2016), Faust et al. (2020), and Karabayir et al. (2022) were recommended by Chen et al. (2023), which consisted of 12, 17, and 541 predictors, respectively, including age and smoking status. The following non-motor symptoms: daytime sleepiness and cognitive impairment were included in Karabayir et al. (2022), urinary dysfunction, constipation and depression were included in Mahlknecht et al. (2016) and Faust et al. (2020) included 536 diagnosis or procedure codes. However, there is still much to learn and uncover on this journey towards a specific diagnostic test for PD. Ultimately, the goal is to develop a highly specific, sensitive, and feasible PD screening biomarker battery. While alpha-synuclein seed amplification assay has shown high sensitivity/specificity in the spinal fluid, the matrix for analysis (CSF) and cost preclude scaling for general population screening. As such clinical screening remains of paramount significance.

Here, we aimed to investigate whether any of the ready clinical markers of PD, such as motor and non-motor symptoms measured with the Movement Disorder Society Unified Parkinson’s Disease Rating Scale (MDS-UPDRS), might be highly specific of PD (common in PD but rare in an age-matched healthy population). This is key as many of the currently used clinical markers of prodromal PD are also common in comparably aged healthy individuals (Pfeiffer, 2016), including depression, sleep problems and hyposmia (Pellicano et al., 2007). Clinical markers specific to prodromal PD (i.e., common in prodromal PD and PD but rare in the comparably aged healthy population) could be used to identify people at high risk of developing PD in the screening of general populations.

We used the Parkinson’s Progression Markers Initiative (PPMI) to investigate and compare the prevalence of clinical markers of PD in participants with prodromal PD, with PD in comparison to healthy controls. Symptoms at high specificity were defined based on a high odds ratio.

## 2. Methods

### 2.1. Study design

The PPMI is an observational, international multi-center study designed to improve the understanding of PD and enhance the success of new experimental treatments (Marek et al., 2011). One commitment of PPMI is to allow the research community to access publicly available PD study data. The participants in this dataset are followed over the course of 5 years. This PPMI dataset was obtained from the LONI Image data archive.^1^ Demographic information, clinical characteristics and results of clinical tests were downloaded from the PPMI database in January, 2021.

The MDS-UPDRS is a clinical rating scale for Parkinson’s disease (Goetz et al., 2008). It is split into four parts:

- Part I, non-motor experiences of daily living,
- Part II, motor experiences of daily living,
- Part III, motor examination,
- Part IV, motor complications.

Potential clinical markers of PD were defined from both motor and non-motor experiences of daily living by measuring each of the individual items from the MDS-UPDRS Part I questionnaire, in addition to the first three items from the MDS-UPDRS Part II questionnaire, speech difficulties, excessive drooling and swallowing difficulties. We chose these items because they describe patient reported symptoms rather than the functional impact of motor symptoms on the activity of daily living (e.g., dressing, hygiene etc.). Item 1.1 focuses on the patient’s cognitive impairment, 1.2 hallucinations and psychosis, 1.3 depressed mood, 1.4 anxious mood, 1.5 apathy, 1.7 sleep problems (insomnia), 1.8 daytime sleepiness, 1.9 pain and other sensations, 1.10 urinary problems, 1.11 constipation, 1.12 lightheadedness on standing, 1.13 fatigue, 2.1 speech difficulties, 2.2 excessive saliva and drooling, and 2.3 chewing and swallowing difficulties.

To investigate the prevalence of each marker we used baseline data from the PPMI database. In our analysis, we included all the participants from the PPMI database who were labeled as healthy controls, participants with prodromal PD or individuals with PD, who had an enrollment date and who had data available for all 15 markers. The inclusion criteria for each cohort within the PPMI database has been described elsewhere (Marek et al., 2011; Mollenhauer et al., 2019; PPMI, 2023a). Briefly, healthy controls were at least 30 years old, had no current or active clinically significant neurological disorder at baseline, and no first-degree relatives diagnosed with PD. The PD cohort consists of participants aged 30 years or older, with a recent clinical diagnosis of PD who are drug naive at baseline and who had a positive dopamine transporter (DAT) single-photon emission computed tomography. The prodromal PD cohort included volunteers aged 60 years or older, with rapid eye movement (REM) sleep behavior disorder (RBD) confirmed by polysomnography, clinically diagnosed by the site investigator, or with hyposmia based on the University of Pennsylvania Smell Identification Test, with DAT deficit. All inclusion and exclusion criteria are noted in the study protocol (PPMI, 2023b).

In order to calculate the prevalence of each marker, the MDS-UPDRS scale was categorized into two groups: symptom present or not: a score of “1–4” implied the presence of the symptom, whereas a score of ‘0’ indicated that the symptom was not present.

### 2.2. Statistical methods

The standardized mean difference (SMD) in baseline and demographic characteristics was used to assess differences between baseline data for the prodromal and healthy controls, PD and healthy controls, and for the prodromal PD and PD participant groups. The SMD was calculated as the absolute value in the difference in means of a covariate across the population groups, divided by the pooled standard deviation (SD). SMDs larger than 0.25 indicate that the populations were different from one another in that variable (Stuart et al., 2013; Pagano et al., 2021). Differences in symptom prevalence could be caused by an imbalance of baseline characteristics across the populations. If that is the case, such baseline characteristics should be accounted for in the model.

To account for the imbalance in baseline characteristics between the three cohorts, we used logistic regression to calculate adjusted odds ratios for each symptom (Sperandei, 2014). The dependent binary variable was the population cohort (HC, Prodromal or PD) and the independent covariates were the symptom, age and gender. A separate model was calculated for each symptom and for each cohort comparison (prodromal versus healthy controls and PD versus healthy controls). The adjusted odds ratio between two cohorts was obtained by exponentiating the coefficient of the specific symptom. All adjusted odds ratios are reported together with a 95% confidence interval. Specificity of a symptom to each population was determined in terms of the adjusted odds ratio. A symptom was categorized as having “high” specificity if the adjusted odds ratio ≥ 6, “moderate” if the adjusted odds ratio ≥ 3 but < 6, or “low” specificity, if the adjusted odds ratio < 3. The prevalence of each symptom was calculated by adding up all the cases when the symptom was present (score 1–4) and dividing it by the total number of subjects in each population (healthy controls, prodromal PD, PD).

## 3. Results

### 3.1. Patient characteristics

A total of 195 healthy controls, 64 prodromal PD and 422 PD individuals were included in the analysis from the PPMI database. The baseline characteristics of the PPMI groups are displayed in Table 1.

**Table 1:** Demographics and baseline characteristics of PPMI participants.

Baseline characteristic	HCs (*n* = 195)	Prodromal (*n* = 64)	PD (*n* = 422)	SMD (95% C.I.) Prodromal vs HC	SMD (95% C.I.) PD vs HC	SMD (95% C.I.) Prodromal vs PD
Mean age (SD)	60.84 (11.26)	68.97 (5.80)	61.65 (9.68)	0.80 (0.51–1.09)	0.08* (−0.09–0.25)	−0.79 (−1.06 to −0.52)
Gender: men (%)	125 (64.1)	50 (78.12)	277 (65.64)	0.14* (0.02–0.26)	0.02* (−0.07–0.10)	−0.12* (−0.24 to −0.01)
Race: white (%)	180 (92.31)	39 (60.94)	388 (91.94)	−0.30 (−0.4 to −0.2)	0.00* (−0.04–0.04)	0.30 (0.18–0.42)
Hispanic/latino	3 (1.54)	20 (31.25)	9 (2.13)	0.31 (0.19–0.42)	0.01* (−0.02–0.03)	−0.30 (−0.42 to –0.19)
American Indian/Alaska native	0 (0)	1 (1.56)	4 (0.95)	0.02* (−0.01–0.05)	0.01* (0.00–0.02)	−0.01* (−0.04–0.03)
Black/African American	10 (5.13)	2 (3.12)	7 (1.66)	−0.02* (−0.07–0.03)	−0.03* (−0.07–0.00)	−0.02* (−0.06–0.03)
Asian	1 (0.51)	0 (0)	10 (2.37)	−0.01* (−0.02–0.00)	0.02* (0.00–0.04)	0.02* (0.01–0.04)
Not Specified	1 (0.51)	2 (3.12)	4 (0.95)	NA	NA	NA
Mean time since diagnosis in months (SD)	NA	NA	6.53 (6.46)	NA	NA	NA
Hoehn and Yahr stage: 0 (%)	193 (98.97)	61 (95.31)	0 (0)	−0.04* (−0.09–0.02)	−0.99 (−1.00 to −0.98)	−0.95 (−1.00 to −0.90)
1	2 (1.03)	2 (3.12)	185 (43.84)	0.02* (−0.02–0.07)	0.43 (0.38–0.48)	0.41 (0.34–0.47)
2	0 (0)	1 (1.56)	235 (55.69)	0.02* (−0.01–0.05)	0.56 (0.51–0.60)	0.54 (0.48–0.60)
3	0 (0)	0 (0)	2 (0.47)	NA	0.00* (0.00–0.01)	0.00* (0.00–0.01)
Mean MDS-UPDRS part I (SD)	2.95 (2.96)	6.36 (3.92)	5.57 (4.07)	1.06 (0.76–1.35)	0.70 (0.52–0.87)	−0.20* (−0.46–0.07)
Mean MDS-UPDRS part II (SD)	0.46 (1.02)	2.14 (2.54)	5.90 (4.19)	1.09 (0.79–1.39)	1.55 (1.36–1.74)	0.94 (0.67–1.21)
Mean MDS-UPDRS part III (SD)	1.21 (2.19)	3.84 (3.81)	20.88 (8.86)	0.98 (0.69–1.28)	2.64 (2.42–2.87)	2.03 (1.74–2.33)
Mean Total MDS-UPDRS (SD)	4.56 (4.4)	12.34 (7.76)	32.35 (13.14)	1.43 (1.13–1.74)	2.49 (2.27–2.71)	1.59 (1.31–1.87)

PD, Parkinson’s disease; HC, healthy controls; SD, standard deviation; SMD, standardized mean difference; C.I., confidence intervals; MDS-UPDRS, movement disorder society-unified Parkinson’s disease rating scale. *SMD within the range of (−0.25, 0.25).

#### 3.1.1. Prodromal vs. healthy controls

Prodromal participants were on average older than the healthy controls [Mean (SD)] [68.9 (5.8) years vs. 60.8 (11.2) years respectively; SMD: 0.8]. The proportion of Caucasians was higher in the healthy control group (92.3%) than in the prodromal group (60.9%). However more Hispanics/Latinos were observed in the prodromal group than in the healthy controls (31.25% vs. 1.54% respectively). The prodromal group had higher progression of disease at baseline with mean (SD) MDS-UPDRS Part I 6.36 (3.92), MDS-UPDRS Part II 2.14 (2.54), MDS-UPDRS Part III 3.84 (3.81), and MDS-UPDRS Total (Sum Part I + II + III) 12.34 (7.76) (Supplementary Figure S1).

#### 3.1.2. PD vs. healthy controls

The majority of the healthy controls were on Hoehn and Yahr Stage 0 (98.9%), whereas the PD participants were distributed among Hoehn and Yahr Stages 1 and 2 (45.8% and 55.6% respectively). The PD group had higher progression of disease at baseline with mean (SD) MDS-UPDRS Part I 5.57 (4.07), MDS-UPDRS Part II 5.90 (4.19), MDS-UPDRS Part III 20.88 (8.86), and MDS-UPDRS Total 32.35 (13.14). All other baseline characteristics were balanced between PD and healthy control groups (Supplementary Figure S2).

#### 3.1.3. Prodromal vs. PD

Prodromal participants were on average older than the PD participants [68.9 (5.8) years vs. 61.6 (9.6) years respectively; SMD: −0.79]. Almost all the participants in the prodromal group were in Hoehn and Yahr Stage 0 (95.3%). MDS-UPDRS Part I was balanced among the two groups (SMD: −0.20). However the PD group presented higher mean values of MDS-UPDRS Part II (SMD: 0.94), MDS-UPDRS Part III (SMD: 2.03), and MDS-UPDRS Total (SMD: 1.59) (Supplementary Figure S3).

### 3.2. Specificity of clinical markers of PD

**Figure 1:**
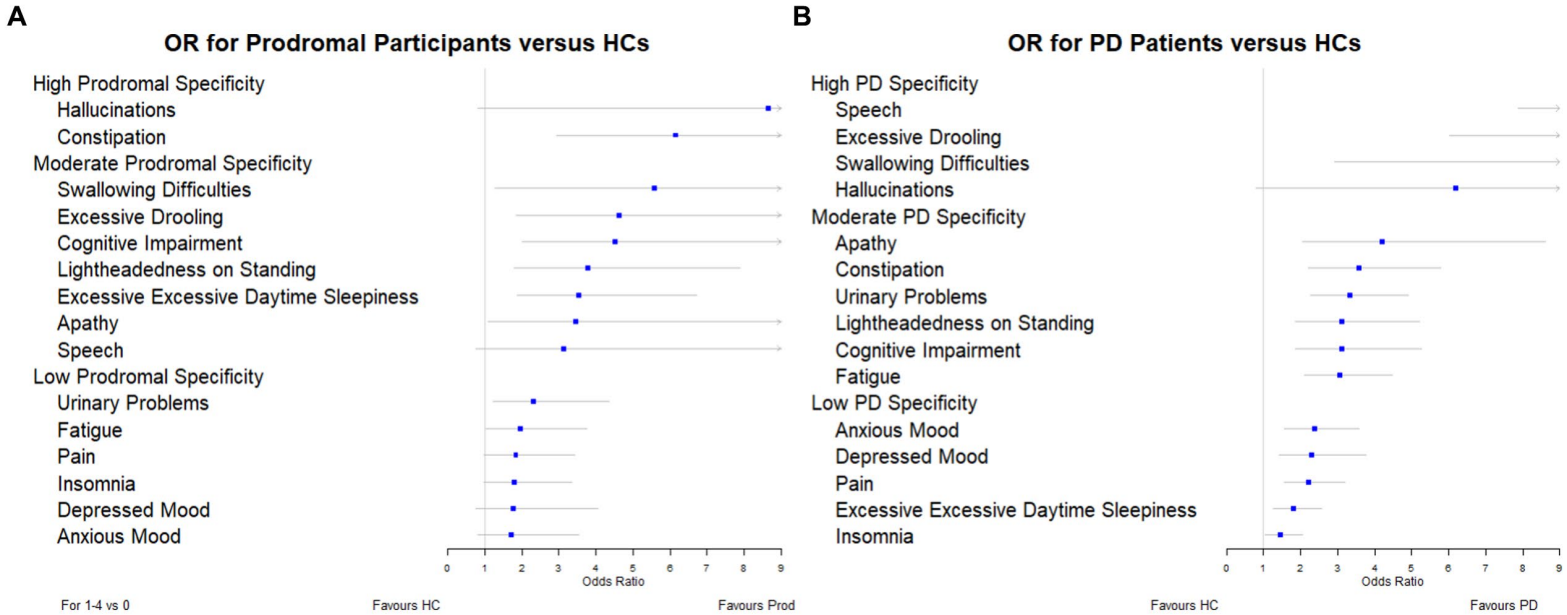
Forest plots showing the adjusted odds ratios for each symptom: **(A)** prodromal participants versus healthy controls, and **(B)** PD individuals versus healthy controls.

To determine which symptoms were more specific to each of the groups under investigation (prodromal PD and PD); we ordered the odds ratios and plotted them in a forest plot (Figure 1). The symptoms classified as highly specific for prodromal PD were visual hallucinations (odds ratio, 8.64 [95% CI: 0.83–90.45]) and constipation (odds ratio, 6.14 [95% CI: 2.94–12.80]). For PD individuals, speech difficulties (odds ratio, 19.61 [95% CI: 7.88–48.81]), excessive drooling (odds ratio, 12.81 [95% CI: 6.04–24.59]), swallowing difficulties (odds ratios, 9.51 [95% CI: 2.94–30.82]) and visual hallucinations (odds ratio, 6.19 [95% CI: 0.81–47.62]) were highly specific. However, the prevalence of visual hallucinations is very small for all three populations, particularly the healthy controls (see Table 2). This is why their adjusted odds ratios are so large, they have very large confidence intervals and they do not have a significant *p*-value for either comparison. Due to these small prevalence, we must be careful when drawing conclusions.

**Table 2:** Adjusted odds ratios and prevalence of each symptom.

Symptom	OR: prodromal PD vs HC (95% C.I.)	*p*-value of symptom in LR	OR: PD vs HC (95% C.I.)	*p*-value of symptom in LR	Prev in HCs	Prev in prodromal PD	Prev in PD
Hallucinations	8.64 (0.83–90.45)	0.07	6.19 (0.81–47.62)	0.08	0.51% (1/195)	4.69% (3/64)	3.08% (13/422)
Constipation	6.14 (2.94–12.80)	<0.01	3.58 (2.22–5.78)	<0.01	12.31% (24/195)	43.75% (28/64)	33.18% (140/422)
Swallowing difficulties	5.57 (1.28–24.12)	0.02	9.51 (2.94–30.82)	<0.01	1.54% (3/195)	9.38% (6/64)	13.03% (55/422)
Excessive drooling	4.62 (1.86–11.45)	<0.01	12.81 (6.04–24.59)	<0.01	4.62% (9/195)	26.56% (17/64)	36.49% (154/422)
Cognitive impairment	4.51 (2.02–10.08)	<0.01	3.12 (1.85–5.26)	<0.01	9.74% (19/195)	29.69% (19/64)	25.36% (107/422)
Lightheaded-ness on standing	3.77 (1.80–7.90)	<0.01	3.12 (1.87–5.20)	<0.01	10.26% (20/195)	35.94% (23/64)	26.3% (111/422)
Excessive daytime sleepiness	3.55 (1.88–6.71)	<0.01	1.82 (1.28–2.58)	<0.01	34.87% (68/195)	65.63% (42/64)	49.53% (209/422)
Apathy	3.45 (1.10–10.83)	0.03	4.20 (2.05–8.60)	<0.01	4.62% (9/195)	10.94% (7/64)	16.82% (71/422)
Speech difficulties	3.12 (0.76–12.73)	0.11	19.61 (7.88–48.81)	<0.01	2.56% (5/195)	7.81% (5/64)	33.89% (143/422)
Urinary problems	2.30 (1.22–4.35)	0.01	3.34 (2.26–4.92)	<0.01	24.10% (47/195)	53.13% (34/64)	50.95% (215/422)
Fatigue	1.96 (1.03–3.75)	0.04	3.06 (2.10–4.47)	<0.01	25.13% (49/195)	42.19% (27/64)	50.24% (212/422)
Pain	1.83 (0.98–3.42)	0.06	2.23 (1.56–3.18)	<0.01	33.85% (66/195)	48.44% (31/64)	52.37% (221/422)
Insomnia	1.80 (0.97–3.34)	0.06	1.46 (1.03–2.05)	0.03	43.59% (85/195)	60.94% (39/64)	53.08% (224/422)
Depressed mood	1.77 (0.77–4.06)	0.18	2.31 (1.42–3.77)	<0.01	12.31% (24/195)	18.75% (12/64)	23.70% (100/422)
Anxious mood	1.70 (0.82–3.54)	0.16	2.38 (1.58–3.59)	<0.01	19.49% (38/195)	26.56% (17/64)	35.78% (151/422)

OR, odds ratio; PD, Parkinson’s disease; HC, healthy controls; C.I., confidence intervals; LR, logistic regression; Prev, prevalence.

### 3.3. Prevalence of clinical markers of PD

**Figure 2:**
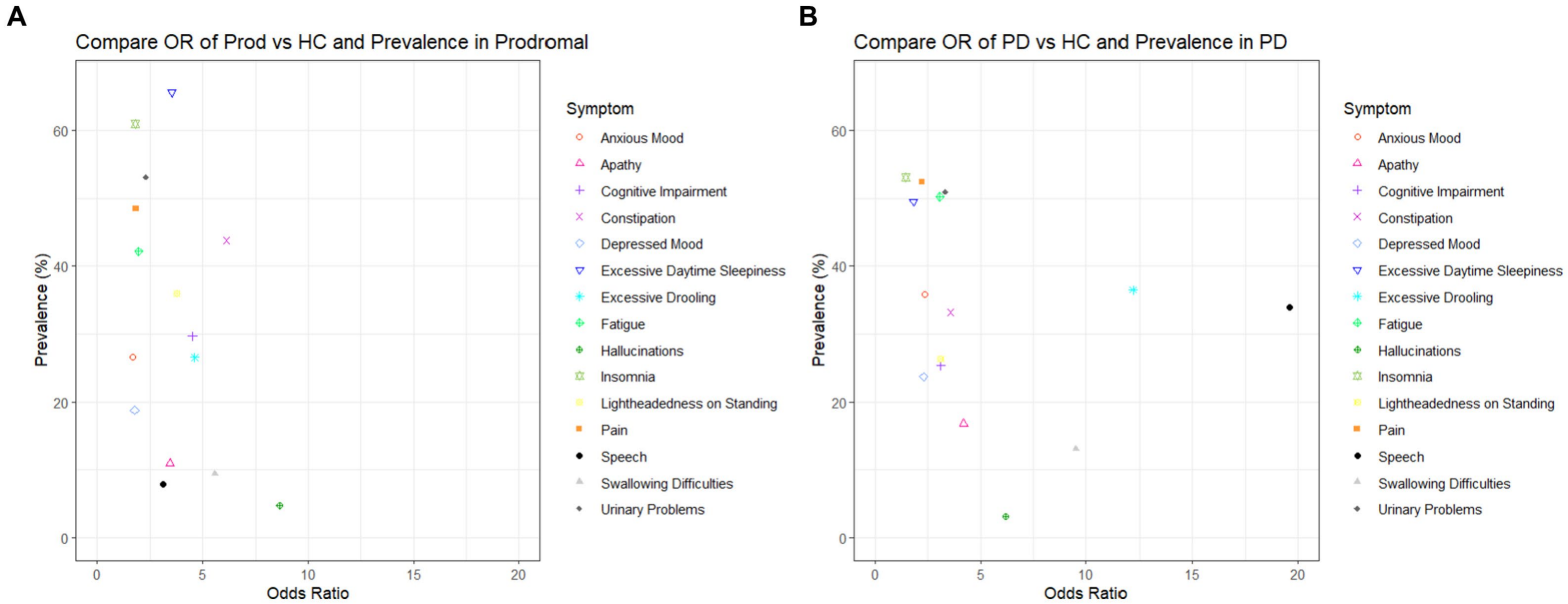
Plots to show the adjusted odds ratio and the prevalence of each symptom when: **(A)** healthy controls are compared to prodromal participants and **(B)** healthy controls are compared to PD patients.

We further investigated the prevalence of each symptom in individuals with prodromal PD and PD (Table 2). Prevalence was plotted against the odds ratio of each symptom in Figure 2. The three symptoms with the largest prevalence in prodromal PD individuals were excessive daytime sleepiness (65.63%), insomnia (60.94%) and urinary problems (53.13%). For individuals with PD, the most prevalent symptoms were insomnia (53.08%), pain (52.37%), and urinary problems (50.95%). These symptoms were among the five most common in healthy controls, with insomnia having a prevalence of 43.59%, excessive daytime sleepiness, 34.87%, pain, 33.85%, and urinary problems, 24.10%. Therefore, these symptoms did not have large adjusted odds ratios for either comparison, as they were also common in healthy controls.

## 4. Discussion

In this study, we investigated the prevalence of symptoms in the prodromal PD population, PD population and in healthy controls. Furthermore, we assessed how specific these symptoms were to individuals with prodromal PD and PD compared to healthy controls, by classifying them as having either: low (adjusted odds ratio < 3), moderate (3 ≤ adjusted odds ratio < 6) or high (6 ≤ adjusted odds ratio) specificity. We observed that visual hallucinations and constipation were the symptoms which were highly specific to prodromal PD. However, of these symptoms, only constipation was prevalent in participants with prodromal PD. Additionally, the symptoms that were highly specific to PD were speech difficulties, excessive drooling, swallowing difficulties and visual hallucinations. Speech difficulties and excessive drooling were prevalent in individuals with PD, whereas swallowing difficulties and visual hallucinations were not.

Of the four most specific symptoms to prodromal PD (those symptoms with the largest odds ratios: visual hallucinations, 8.64, constipation, 6.14, swallowing difficulties, 5.57, excessive drooling 4.62), only constipation and excessive drooling were moderately prevalent in the prodromal PD population, 43.75% and 26.56%. Visual hallucinations and swallowing difficulties were among the least common in the prodromal PD population. Of the four most specific symptoms to PD (those symptoms with the largest odds ratios: speech difficulties, 19.61, excessive drooling, 12.18, swallowing difficulties, 9.51, visual hallucinations, 6.19), only excessive drooling and speech difficulties were moderately prevalent in the PD population, 36.49% and 33.89%. Visual hallucinations and swallowing difficulties were among the least common in the PD population. This indicates that the symptoms that are most specific to the disease (prodromal PD or PD), are not necessarily the symptoms which are most prevalent in each disease population, and vice versa (see Figure 2).

We further found that the clinical markers in prodromal PD and PD were different. This supports the hypothesis that symptoms may develop at different times over the course of the disease. The adjusted odds ratio for speech difficulties gave the largest increase from participants living with prodromal PD to PD, this could indicate that this clinical marker becomes more common as patients progress. Varanese et al. (2010), supports this idea. In contrast, the adjusted odds ratio for visual hallucinations decreased by the largest amount from prodromal PD to PD participants. One reason behind this decrease could be that this symptom becomes less common as patients progress. However, Varanese et al. (2010) suggests the opposite. They suggest that visual hallucinations become more common over the course of the disease. Therefore, an alternative reason may be behind this decrease in adjusted odds ratio. Prodromal PD is also prodromal of dementia with Lewy bodies. Therefore, individuals labeled as prodromal PD within PPMI might not actually develop PD in the future; they may develop dementia with Lewy bodies instead. As these prodromal individuals have a higher risk of developing dementia than the PD cohort, they might also have a larger adjusted odds ratio to suffer from visual hallucinations, which is a key cardinal symptom of dementia with Lewy bodies.

It is generally thought that visual hallucinations, constipation, swallowing difficulties and excessive drooling are symptoms which dominate late stages of PD (Rukavina et al., 2021). This is not in complete contrast to our findings, as we observed a low prevalence of visual hallucinations and swallowing difficulties in prodromal PD participants. However, whereas many previous studies and articles explore the prevalence of symptoms in the prodromal PD and PD population, they do not often compare the prevalence of these symptoms to the age-matched healthy controls. Even though visual hallucinations and swallowing difficulties are not common in participants living with prodromal PD, they may still be specific symptoms of prodromal PD as their prevalence was much higher in prodromal PD compared to the healthy control population.

In order for a symptom to aid in the early diagnosis of prodromal PD, it should be both highly specific and sufficiently prevalent in the prodromal PD population to detect enough participants. However, we have shown above that the symptoms that are most specific to the disease (prodromal PD or PD), are not necessarily the symptoms which are most prevalent in these disease populations, and vice versa. Unfortunately, there are not many symptoms which are both, so one must compromise to select symptoms which are either moderately specific and highly prevalent or highly specific and only moderately prevalent. Therefore, we suggest it is the symptoms, such as constipation and excessive drooling, which had large and moderate adjusted odds ratios (6.14 and 4.62), moderately high prevalence in prodromal participants (43.75% and 26.56%) and fairly low prevalence in healthy controls (12.31% and 4.62%), which would make robust predictors of prodromal PD. In addition, symptoms such as excessive drooling and speech difficulties, which had large adjusted odds ratios (12.81 and 19.61), moderately high prevalence in PD patients (36.49 and 33.89%) and low prevalence in healthy controls (4.62% and 2.56%), which would make robust predictors of PD (see Table 2).

The main strength of this study is the comparison of all three cohorts: healthy controls, prodromal PD participants, as well as individuals with PD. Today, prodromal PD is particularly difficult to detect (Berg et al., 2014). Therefore, these individuals are not included in many studies or even if they are, they are not deeply characterized with information with every item of the MDS-UPDRS. Due to the lack of treatment to delay PD progression, it is particularly important to try to diagnose PD earlier in patients and recruit these early PD patients into clinical trials of drugs designed to slow the progression of the disease. The comparison of healthy controls and prodromal PD participants in this work, allows us to detect which symptoms could be used in future to enable this earlier PD diagnosis. Furthermore, many studies look at the prevalence of symptoms in PD patients only and do not include a healthy control comparison. This comparison is particularly useful, as many symptoms, which are most common in PD patients (such as insomnia, pain, and urinary problems) are also common in age-matched healthy controls. With these two comparisons, we can see which symptoms are most common in people suffering from prodromal PD compared to healthy controls and see how their prevalence develops as prodromal PD participants progress and develop clinical PD. Prior studies have demonstrated the high risk of PD onset in the prodromal PD population with hyposmia or RBD (Iranzo et al., 2017; Jennings et al., 2017). Pilot prodromal data from PPMI indicate that 35% of the prodromal PD participants with hyposmia or RBD with abnormal DAT developed PD within the first four years (PPMI, 2023b). We expect this number to increase as time continues.

There are potential limitations of this study. Firstly, in the PPMI study, the prodromal PD cohort was a pilot effort, as such, the sample size of this population is relatively small compared to the numbers of healthy controls and individuals with PD recruited. This causes some of the confidence intervals, especially for symptoms such as visual hallucinations, excessive drooling, swallowing difficulties and speech difficulties, to be particularly wide. Secondly, the prevalence of these symptoms in the healthy control population may not be generalizable to the healthy control population outside of PPMI (Jackson et al., 2022). Additionally, the prodromal PD and PD cohorts had different eligibility criteria (Mollenhauer et al., 2019). Only participants with isolated RBD or isolated hyposmia were recruited into the prodromal PD cohort investigated here. The PD participants needed to be older than 30, whereas the prodromal PD participants had to be at least 60 years old. In addition, the PD patients were required to have a DaT deficit in the putamen on 123-I Ioflupane DaT imaging, conversely, only 80% of the prodromal PD participants had a similar DaT deficit (Mollenhauer et al., 2019). Furthermore, the majority of the prodromal PD cohort suffered from isolated rapid eye movement sleep behavior disorder and as such, were likely to develop dementia with Lewy bodies with visual hallucinations. Due to the characteristics of the prodromal PD cohort within the PPMI database, the dementia with Lewy bodies phenoconversions are driving some important symptom differences. Therefore, one must be careful when making conclusions about the differences between the prodromal PD and PD populations. In addition, we only looked at the 15 symptoms available in the MDS-UPDRS, there are other tools available which track more symptoms (and in greater detail) in PD participants. These include the non-motor symptom questionnaire (Chaudhuri et al., 2006) and the non-motor symptom scale (Chaudhuri et al., 2007), which evaluate a further 15 symptoms, or the more recent MDS version of the non-motor symptom scale (Martinez-Martin et al., 2019). These scales would have been useful if they had been included in the PPMI database. As this is an exploratory study by nature, we make no corrections for multiplicity.

Further work includes correlating clinical markers with DaT binding and performing subgroup analysis with clustering of prodromal markers (RBD, hyposmia and DaT deficit). Unfortunately, this is not possible yet within PPMI for external investigators (PPMI, 2023c). In addition, these results must be validated in another dataset or multiple datasets, which have a larger and more generalizable prodromal PD and healthy control cohort. Next steps would be performing a meta-analysis of all published studies including motor and non-motor features of PD to confirm our hypothesis in a much larger sample size. In addition, when the most useful symptoms, which could aid in the early diagnosis of prodromal PD have been identified, the next step would be to link these symptoms to biomarkers. If these useful symptoms can be anchored to a certain biomarker (or biomarkers), then these biomarkers could be used to aid in the diagnosis of prodromal PD even earlier.

## Data availability statement

Publicly available datasets were analyzed in this study. This data can be found here: https://ida.loni.usc.edu/login.jsp. This PPMI dataset was obtained from the LONI Image data archive in January 2021.

## Ethics statement

Ethical review and approval was not required for the study of human participants in accordance with the local legislation and institutional requirements.

## Author contributions

GP and JA-C designed the study. HJ and JA-C were involved in data collection and analyzing the data. GP, JA-C, and HJ contributed to data interpretation. All authors contributed to the article and approved the submitted version.

## Footnotes

^1^https://ida.loni.usc.edu/login.jsp
