# Supplementary material for: Identifying prodromal symptoms at high specificity for Parkinson’s disease
Source: Front Aging Neurosci. 2023 Sep 22;15:1232387. doi: 10.3389/fnagi.2023.1232387 (PMC10556459; doi:10.3389/fnagi.2023.1232387)
Supplement: Supplementary file 1 [file Data_Sheet_1.pdf]

# Identifying prodromal symptoms at high specificity for Parkinson's disease

## Supplementary material

**Figure S1:** Covariate balance (Love plot) between Healthy Controls and Prodromal Participants

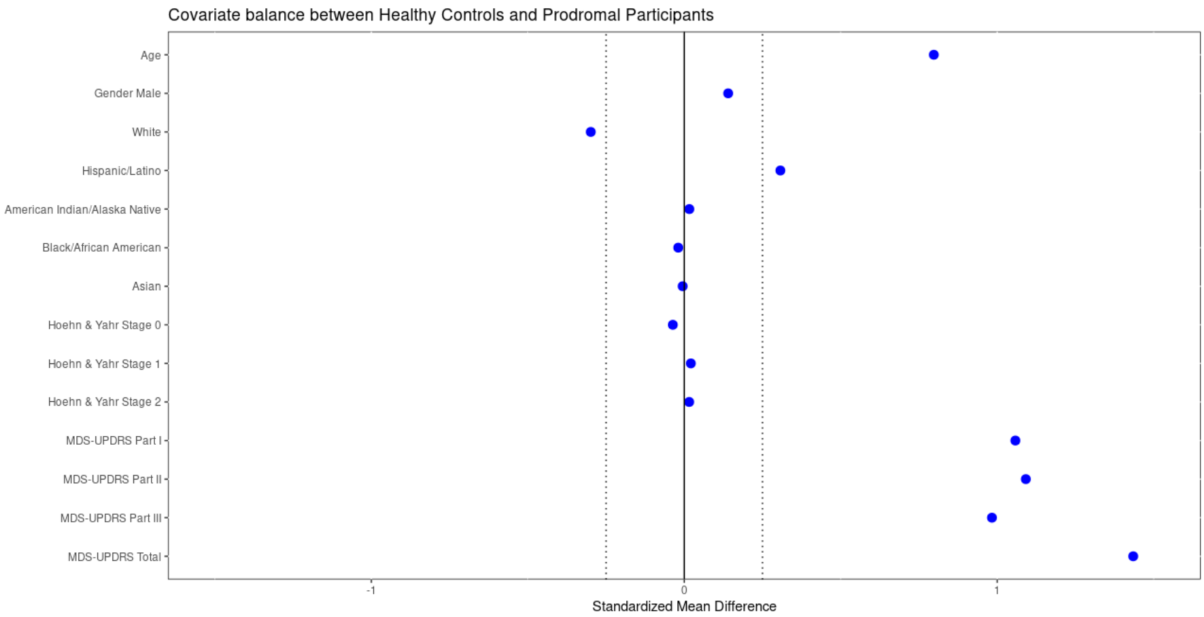


MDS-UPDRS, Movement Disorder Society – Unified Parkinson's Disease Rating Scale. Continuous variables display standardized mean difference (SMD). Binary variables display differences in proportions.

**Figure S2:** Covariate balance (Love plot) between Healthy Controls and Parkinson's Disease Participants

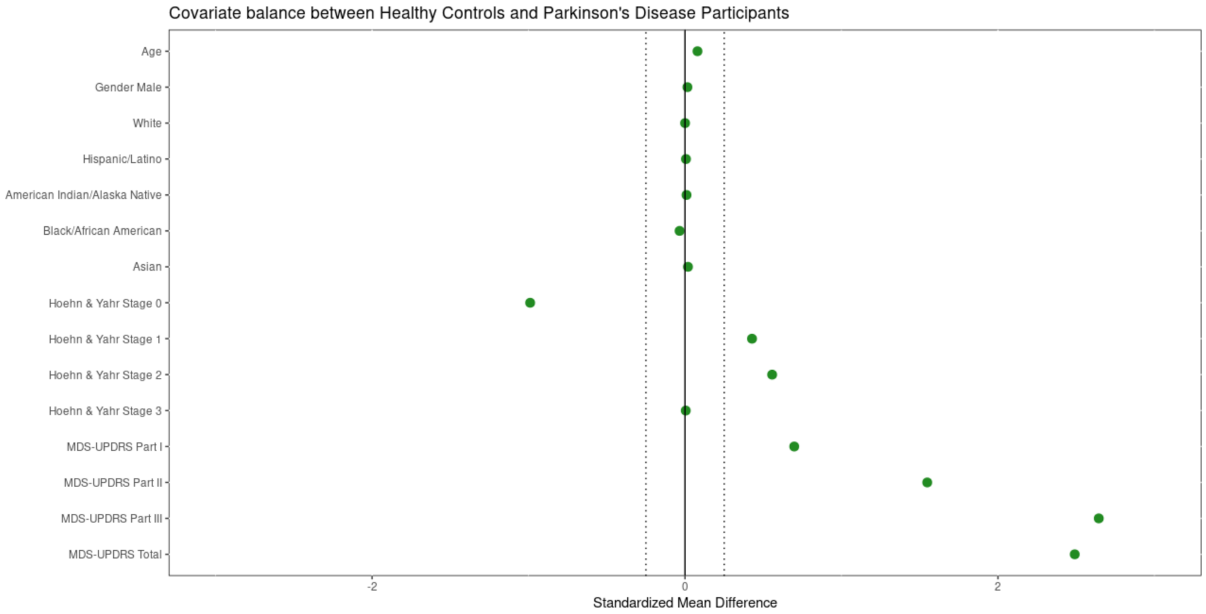


MDS-UPDRS, Movement Disorder Society – Unified Parkinson's Disease Rating Scale. Continuous variables display standardized mean difference (SMD). Binary variables display differences in proportions.

**Figure S3:** Covariate balance (Love plot) between Prodromal and Parkinson's Disease Participants

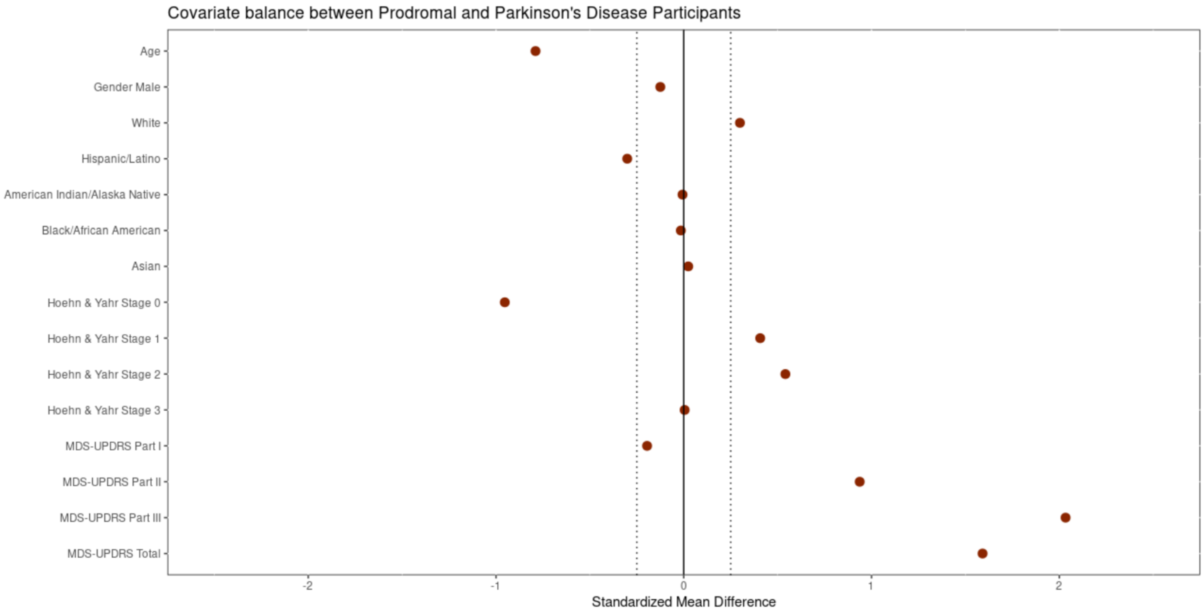


MDS-UPDRS, Movement Disorder Society – Unified Parkinson's Disease Rating Scale. Continuous variables display standardized mean difference (SMD). Binary variables display differences in proportions.
